# High-dose paclitaxel in combination with doxorubicin, cyclophosphamide and peripheral blood progenitor cell rescue in patients with high-risk primary and responding metastatic breast carcinoma: toxicity profile, relationship to paclitaxel pharmacokinetics and short-term outcome

**DOI:** 10.1054/bjoc.2001.1835

**Published:** 2001-06

**Authors:** G Somlo, J H Doroshow, T Synold, J Longmate, D Reardon, W Chow, S J Forman, L A Leong, K A Margolin, R J Morgan Jr, J W Raschko, S I Shibata, M L Tetef, Y Yen, N Kogut, J Schriber, J Alvarnas

**Affiliations:** Departments of Medical Oncology and Therapeutics Research and Biostatistics, City of Hope Comprehensive Cancer Center, Duarte, CA 91010; City of Hope Bone Marrow Transplant Unit/The Good Samaritan Hospital, Phoenix, AZ 85006; Regional Bone Marrow Transplantation Program, Southern California Kaiser Permanente, Los Angeles, CA, 90027, USA

## Abstract

We assessed the feasibility and pharmacokinetics of high-dose infusional paclitaxel in combination with doxorubicin, cyclophosphamide, and peripheral blood progenitor cell rescue. Between October 1995 and June 1998, 63 patients with high-risk primary [stage II with ≥ 10 axillary nodes involved, stage IIIA or stage IIIB inflammatory carcinoma (*n* = 53)] or with stage IV responsive breast cancer (*n* = 10) received paclitaxel 150–775 mg/m^2^infused over 24 hours, doxorubicin 165 mg/m^2^as a continuous infusion over 96 hours, and cyclophosphamide 100 mg kg^–1^. There were no treatment-related deaths. Dose-limiting toxicity was reversible, predominantly sensory neuropathy following administration of paclitaxel at the 775 mg/m^2^dose level. Paclitaxel pharmacokinetics were non-linear at higher dose levels; higher paclitaxel dose level, AUC, and peak concentrations were associated with increased incidence of paraesthesias. No correlation between stomatitis, haematopoietic toxicities, and paclitaxel dose or pharmacokinetics was found. Kaplan–Meier estimates of 30-month event-free and overall survival for patients with primary breast carcinoma are 65% (95% CI; 51–83%) and 77% (95% CI; 64–93%). Paclitaxel up to 725 mg/m^2^infused over 24 hours in combination with with doxorubicin 165 mg/m^2^and cyclophosphamide 100 mg kg^–1^is tolerable. A randomized study testing this regimen against high-dose carboplatin, thiotepa and cyclophosphamide (STAMP V) is currently ongoing. © 2001 Cancer Research Campaign http://www.bjcancer.com


*British Journal of Cancer* (2001) **84**(12), 1591–1598
© 2001 Cancer Research Campaign
doi: 10.1054/ bjoc.2001.1835, available online at http://www.idealibrary.com on IDEAL®

http://www.bjcancer.com

### High-dose paclitaxel in combination with doxorubicin, cyclophosphamide and peripheral blood progenitor cell rescue in patients with high-risk primary and responding metastatic breast carcinoma: toxicity profile, relationship to paclitaxel pharmacokinetics and short-term outcome

**G Somlo[1], JH Doroshow[1], T Synold[1], J Longmate[1], D Reardon[1], W Chow[1], SJ Forman[1], LA Leong[1], KA Margolin[1], RJ Morgan Jr[1], JW Raschko[1], SI Shibata[1], ML Tetef[1], Y Yen[1], N Kogut[3], J Schriber[2] and J Alvarnas[2]**

[1]Departments of Medical Oncology and Therapeutics Research and Biostatistics, City of Hope Comprehensive Cancer Center, Duarte, CA 91010; [2]City of Hope Bone Marrow Transplant Unit/The Good Samaritan Hospital, Phoenix, AZ 85006; [3]Regional Bone Marrow Transplantation Program, Southern California Kaiser Permanente, Los Angeles, CA, 90027 USA

**Summary** We assessed the feasibility and pharmacokinetics of high-dose infusional paclitaxel in combination with doxorubicin, cyclophosphamide, and peripheral blood progenitor cell rescue. Between October 1995 and June 1998, 63 patients with high-risk primary [stage II with ≥ 10 axillary nodes involved, stage IIIA or stage IIIB inflammatory carcinoma ($n$ = 53)] or with stage IV responsive breast cancer ($n$ = 10) received paclitaxel 150–775 mg/m$^2$ infused over 24 hours, doxorubicin 165 mg/m$^2$ as a continuous infusion over 96 hours, and cyclophosphamide 100 mg kg$^{-1}$. There were no treatment-related deaths. Dose-limiting toxicity was reversible, predominantly sensory neuropathy following administration of paclitaxel at the 775 mg/m$^2$ dose level. Paclitaxel pharmacokinetics were non-linear at higher dose levels; higher paclitaxel dose level, AUC, and peak concentrations were associated with increased incidence of paraesthesias. No correlation between stomatitis, haematopoietic toxicities, and paclitaxel dose or pharmacokinetics was found. Kaplan–Meier estimates of 30-month event-free and overall survival for patients with primary breast carcinoma are 65% (95% CI; 51–83%) and 77% (95% CI; 64–93%). Paclitaxel up to 725 mg/m$^2$ infused over 24 hours in combination with with doxorubicin 165 mg/m$^2$ and cyclophosphamide 100 mg kg$^{-1}$ is tolerable. A randomized study testing this regimen against high-dose carboplatin, thiotepa and cyclophosphamide (STAMP V) is currently ongoing. © 2001 Cancer Research Campaign http://www.bjcancer.com

The role of high-dose chemotherapy and peripheral blood progenitor cell rescue (HDCT) for responding metastatic or high-risk primary breast cancer (HRBC) has been under evaluation for over decade (Williams et al, 1989; Antman et al, 1992, 1997; Peters et al, 1993). Two small randomized studies could not confirm the benefit of adjuvant HDCT (Hortobagyi et al, 1998; Rodenhuis et al, 1998). Preliminary data from a recently completed phase III trial in patients with ≥ 4 axillary nodes involved, suggest relapse-free and overall survival benefit in the first 284 patients, however, this benefit has not yet been confirmed in all 885 patients, therefore it is too soon to draw definite conclusions from this study (Rodenhuis et al, 2000). Preliminary data from a phase III US trial in HRBC suggested a decreased relapse rate, but at the cost of unacceptably high treatment-related mortality following HDCT with BCNU, cisplatin and cyclophosphamide (Peters et al, 1999). In partially responsive stage IV breast cancer equivalent outcome was observed with high-dose cyclophosphamide, thiotepa and carboplatin (STAMP V) versus a median of 8 cycles of cyclophosphamide, methotrexate and 5-fluoruracil (CMF); no conclusion regarding the potential benefit of HDCT in patients in complete remission was reached in this study (Stadtmauer et al, 2000).

*Received 11 September 2000*
*Revised 19 February 2001*
*Accepted 19 March 2001*

*Correspondence to:* G Somlo

Doxorubicin doses of 75–150 mg/m$^2$ yield an 80% response rate in stage IV breast cancer (Bronchud et al, 1989); low doses of adjuvant doxorubicin (< 40 mg/m$^2$/cycle) are associated with diminished relapse-free survival, although no additional benefit from moderately higher doses of doxorubicin (60 mg/m$^2$ versus 40 mg/m$^2$) was observed (Wood et al, 1994). We previously tested a doxorubicin-containing HDCT regimen (doxorubicin 165 mg/m$^2$, etoposide 60 mg kg$^{-1}$, and cyclophosphamide 100 mg kg$^{-1}$ (CAVP) and observed an encouraging progression-free survival of 69% in patients with HRBC at 4.5 years; treatment-related mortality was under 1% with mucositis as the most significant toxicity; a projected ~20% progression-free survival in patients with responsive stage IV disease was also seen (Somlo et al, 1993, 1997a; Doroshow et al, 1995). To replace the least effective agent, etoposide, and to improve toxicity profile, we developed a novel HDCT combination, inclusive of paclitaxel (Taxol®(T)), a drug with reported response rates of 56% to 62% in patients with metastatic breast cancer (Holmes et al, 1991; Reichman et al, 1993). Preclinical data favour prolonged adminstration of T; increased dose/exposure enhances apoptosis (O'Shaughnessy et al, 1994; McCloskey et al, 1996). In the clinical setting, 24-hour infusion of T results in higher response rates in stage IV breast cancer than a 3-hour infusion (Mamounas et al, 1998).

Here, we report on the feasibility of combining 24-hour infusional T with high-dose doxorubicin and cyclophosphamide (ACT) and describe the pharmacokinetics of T and doxorubicin and their effect on toxicity. Preliminary data on clinical outcome are also reported.

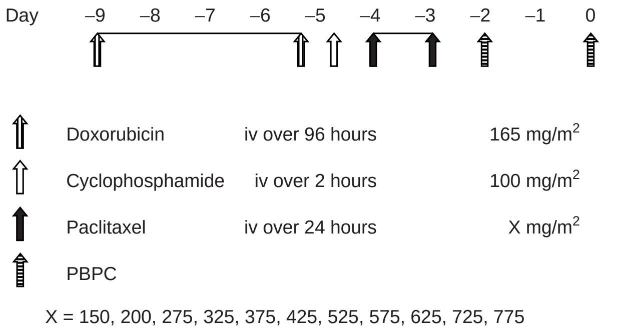

**Figure 1** High-dose chemotherapy schema and paclitaxel dose escalation

### MATERIALS AND METHODS

### Eligibility

This study was approved by the Institutional Review Board of the City of Hope National Medical Center. All patients participating in this trial gave written, voluntary informed consent.

Patients with HRBC (stage II with ≥ 4 axillary nodes involved, stage IIIA, or stage IIIB carcinoma) or with responsive stage IV disease (complete or partial response due to chemotherapy, radiation treatment, or surgery, and confirmed twice at least 4 weeks apart) were eligible. Patients were to start HDCT within 6 months from completion of any standard chemotherapy and must not have received any treatment for at least 4 weeks prior to enrolment on this study. Prior exposure to doxorubicin of ≤ 180 mg/m$^2$, and to paclitaxel of ≤ 750 mg/m$^2$, and for patients with metastatic disease ≤ 2 induction regimens were allowed. Patients who received prior radiation to the left chest wall were excluded. A Karnofsky performance status of ≥ 80%, physiological age of ≤ 60 years, bilirubin within normal range, ASAT and ALAT ≤ 2 x the upper limit of normal, a measured creatinine clearance ≥ 70 ml min$^{-1}$, cardiac left ventricular ejection fraction (LVEF) ≥ 55% as documented by cardiac radionuclide-gated pool scanning (MUGA), a forced expiratory volume in 1 second (FEV1) of ≥ 2 l, and diffusion capacity > 60% of predicted, were required. Computed tomography (or MRI) of the brain, chest, and abdomen, a bone scan, baseline audiograms and nerve conduction studies and bilateral bone marrow biopsies were required. A faculty pathologist reviewed haematoxylin and eosin-stained slides of the primary tumour, samples of metastases and bone marrow biopsies. Patients with bone marrow or CNS metastases were excluded.

### Treatment plan

#### *Stem-cell (PBPC) procurement*

The majority of patients ($n$ = 54) underwent mobilization with G-CSF (Filgrastim, Amgen Inc, Thousand Oaks, CA) 5 µg kg$^{-1}$ bid, subcutaneously (sc); on the 5th day of priming apheresis commenced and continued through collection of at least $8 \times 10^8$ mononuclear cells kg$^{-1}$. Nine patients were enrolled on a phase II randomized, placebo-controlled study of recombinant human thrombopoietin (rhTPO), (Genentech Inc, South San Francisco, CA) and/or G-CSF priming and post-reinfusion randomization between placebo, rhTPO and G-CSF or GM-CSF (Immunex, Seattle, WA); for these patients a minimum of $2 \times 10^6$ CD34$^+$ cells kg$^{-1}$ and a target of > $5 \times 10^6$ CD34$^+$ cells kg$^{-1}$ were required. PBPC were collected and were immediately cryopreserved as reported earlier (Somlo et al, 1997a), except for 1 patient who underwent CD34$^+$ selection using an Isolex Magnetic Cell Separator (Nexell Therapeutics Inc, Irvine, CA).

#### *High-dose chemotherapy regimen*

The HDCT schema and T dose escalation are illustrated in Figure 1. Ideal body weight was used when calculating doses. Doxorubicin 165 mg/m$^2$ was administered over 96 hours, between days –9 and –5, followed by cyclophosphamide 100 mg kg$^{-1}$ on day –5, as reported earlier (Somlo et al, 1997b); a 24 hour continuous i.v. infusion of T was started on day –4. 25% of PBPC were reinfused on day –2 and 75% on 0 (Somlo et al, 1997b), except for the 9 patients receiving rhTPO mobilization and 1 patient whose PBPC were selected using the Isolex device; these patients received their PBPC on day 0 and then were given a combination of rhTPO and G-CSF or GM-CSF, or G-CSF alone. Supportive care was provided as per institutional standards; G-CSF 5 µg kg$^{-1}$, bid, i.v. was started with the first stem cell reinfusion and was continued through absolute granulocyte recovery (AGC) of ≥ $1 \times 10^9$ l$^{-1}$ for 3 consecutive days (Somlo et al, 1997a).

### Toxicity grading and dose adjustments

The dose of T was escalated as illustrated in Figure 1. Doxorubicin and cyclophosphamide were administered without any dose modification. We utilized the NCI Division of Cancer Treatment Common Toxicity Criteria for all organ toxicities except for the hematologic and gastrointestinal systems, where we followed guidelines we have incorporated in our prior HDCT trials (Somlo et al, 1994, 1997a). The maximum tolerated dose of this regimen (MTD) was defined as grade 3 toxicity for any system in 2 of 6 patients treated at that dose level after 21 days of observation. The MTD was also to be established at any given dose level if a single patient experienced grade 4 toxicity. Then, we planned to expand the study to incorporate a total of 20 patients for treatment at MTD-1 in order to gain more clinical experience and assess to patient-to-patient variations of T pharmacokinetics.

### Pharmacokinetics

Blood samples were drawn from a peripheral line immediately prior to the start of the doxorubicin infusion, 48, 72 and 96 hours during infusion, and at 2, 6, 12, 24 and 48 hours after completion of the infusion. Plasma doxorubicin and doxorubicinol concentrations were determined using a previously described loop-column method (Riley et al, 1985).

Peripheral blood samples were also obtained immediately prior to the start of the T infusion, at 3, 6, 12, 20 and 24 hours during the T infusion, then at 15 minutes, 30 minutes, 1, 2, 3, 6, 12 and 24 hours after completion of the infusion. The concentration of T in plasma was measured according to a previously published HPLC method (Rizzo et al, 1990). Pharmacokinetic data analyses of individual plasma drug concentration versus time curves were performed using ADAPT II software (Biomedical Simulations Resource, University of Southern California, Los Angeles, CA).

 *© 2001 Cancer Research Campaign*

Doxorubicin and doxorubicinol concentrations were fit simultaneously using a dual 2-compartmental pharmacokinetic model, while T concentrations were fit with a previously described pharmacokinetic model incorporating both saturable distribution and saturable elimination (Sonnichsen et al, 1994; Gianni et al, 1995). Adequacy of the model derived fits were assessed by examination of residuals between predicted and measured T concentrations, along with the correlation coefficients. Doxorubicin, doxorubicinol and T AUCs were estimated using linear trapezoids, with the terminal area extrapolated to infinity, using both the measured and fitted drug concentrations. Doxorubicin and T systemic clearances ($CL_{sys}$) were determined using the relationship: $CL_{sys}$ = dose/AUC. Peak plasma T concentrations were defined as the actual T levels measured just prior to the end of the infusion.

### Post-HDCT management

#### *Radiation therapy*

Radiation therapy to the chest wall and draining lymph nodes at a dose of 40–50 Gy (with boosts of 10–15 Gy to the primary site) was offered to patients with stage II and IIIA/B breast cancer. Patients treated for stage IV disease were to receive local-regional radiation to control metastatic sites, as it was deemed feasible by the treating physicians.

#### *Hormone therapy*

Following HDCT, tamoxifen 10 mg bid was prescribed to patients with receptor positive high-risk breast cancer, or to those with metastatic disease who had previously not failed tamoxifen.

### Response evaluation and post-treatment follow-up

Patients were evaluated at day 30 and subsequently, at least once every 4 months. Yearly mammograms and scans of previously involved areas for patients with metastatic disease, were performed at the time of follow-up, or as clinically indicated. A MUGA scan and audiogram and nerve conduction studies were repeated 30 days following HDCT. Standard response and progression criteria were applied (Somlo et al, 1994, 1997).

### Statistical analysis

Outcomes examined included overall survival (OS) and relapse-free survival (RFS), calculated from the first day of HDCT for HRBC patients. OS and progression-free survival (PFS) in patients with stage IV disease were calculated from the first day of HDCT. The product-limit method of Kaplan–Meier was used when calculating projected survivals (Kaplan and Meier, 1958). Data entry was censored as of 3/3/2000. Other data are summarized as median and range, or as frequencies and percentages, as appropriate. Association of T AUCs, peak concentrations and clearance with toxicities, and in the 20 patients treated at the MTD-1 level (T 725 mg/m$^2$) with body weight, ALAT, ASAT, alkaline phosphatase, bilirubin, and age were evaluated by linear regression, Wilcoxon rank-sum tests, and logistic regression. Significance levels are reported as '$P$ values' and 'significant' denotes $P < 0.05$ when not otherwise specified. Initial data management was done using SAS, and statistical computing was done using SPlus 4.5 release 2.

### RESULTS

### Patients

Between October 1995 and June 1998, 63 patients were entered on study. Table 1 lists patient characteristics. The median age was 46 years (range, 29–61). All patients with primary breast cancer received a doxorubicin-containing adjuvant regimen but only 1 patient received T prior to HDCT. The median length of time between diagnosis and initiating HDCT was 4.9 months (range, 3.6–10.2).

10 patients received HDCT for stage IV disease; all patients received induction chemotherapy (doxorubicin-containing regimen: 40%; doxorubicin and T: 40%; T-containing regimen: 20%). Of 5 patients in complete remission (CR) prior to HDCT, 3 patients underwent surgical removal of their local-regional metastasis (lymph node, skin and chest wall lesions, respectively) first, and proceeded to undergo 'induction' chemotherapy; 2 patients achieved complete remission (CR) with induction therapy alone.

### Pharmacokinetic analysis

Doxorubicin pharmacokinetics were examined in a subset of 6 patients; doxorubicin and doxorubicinol plasma concentrations rose during the entire infusion, reaching a maximum of 50–70 ng ml$^{-1}$ (0.1 μM). After the end of infusion, plasma doxorubicin concentrations declined in a bi-exponential manner. The mean doxorubicin clearance was 28.6 ± 2.4 l /h/m$^2$ and the mean doxorubicin to doxorubicinol AUC ratio was 0.64 ± 0.2 (data not shown). Comparison of the doxorubicin pharmacokinetic data with previously published findings demonstrate that doxorubicin clearance is linear up to the doses used in the current study (Synold and Doroshow, 1996).

Paclitaxel (T) pharmacokinetic data are available from 52 patients at doses ranging from 150 to 775 mg/m$^2$, including 16 patients treated at the MTD-1 of 725 mg/m$^2$. Over the entire dose range, T plasma concentration-versus-time data were best described by a pharmacokinetic model incorporating saturable distribution and clearance. Plasma AUC values ranged from 4.3 to 96.1 μM×h, and $C_{max}$ levels ranged from 2.1 to 52.1 μM. Figure 2 depicts the relationship between T dose and systemic clearance. T clearances ranged from 52.1 to 8.8 1/h/m$^2$, and decreased with dose. As a result, both T AUC and $C_{max}$ increased non-linearly with dose. Figure 3 demonstrates the non-linearity of T AUC versus dose, with the relationship being best described by a quadratic equation ($P$ = 0.001). A non-linear increase in T $C_{max}$ with increasing dose ($P$ = 0.02) was also seen (not shown).

In addition to the disproportionate increases in T AUC and $C_{max}$ with increasing dose, variability in the measured systemic exposure also increased over the entire dosing range ($P < 0.00001$ for AUC and $P$ = 0.0002 for $C_{max}$). Within the group of patients treated at the 725 mg/m$^2$ dose level, T clearances ranged from 8.8 to 32.4 l /h/m$^2$. As a result, T AUC in this cohort varied from 26.2 to 96.1 μM×h. The spread between the lowest and highest AUC and peak concentrations in these patients was 3.7 fold and 2.5 fold, respectively. Similarly wide ranges in AUC (Figure 3) and $C_{max}$ (data not shown) were seen in patients treated with 625 mg/m$^2$, 3.2 and 4.6 fold respectively.

© 2001 Cancer Research Campaign

**Table 1** Patient characteristics

| | *N* | (%) | Median | (Range) |
|---|---|---|---|---|
| Age at diagnosis, years | | | 46 | (29–61) |
| **Patients with stage II/IIIA/IIIB BC** | **53** | **(100)** | | |
| Patients with stage II BC | 27 | (51) | | |
| IIIA/B BC | 26 | (49) | | |
| ER and/or PR positive primary | 38 | (72) | | |
| Treated with modified radical mastectomy | 44 | (83) | | |
| Breast conservation | 9 | (17) | | |
| Prior doxorubicin exposure | 53 | (100) | | |
| Prior T exposure | 1 | (2) | | |
| Prior radiation therapy | 1 | (2) | | |
| Prior tamoxifen therapy | 2 | (4) | | |
| Time from diagnosis to HDCT, months | | | 4.9 | (3.6–10.2) |
| Radiation therapy post-HDCT | 49 | (93) | | |
| Tamoxifen post HDCT | 40 | (76) | | |
| **No. of patients with stage IV BC** | **10** | **100** | | |
| ER and/or PR positive primary | 9 | (90) | | |
| Prior adjuvant A-based regimen | 2 | (20) | | |
| non-A containing regimen | 5 | (50) | | |
| Induction with an A-based regimen± | 4 | (40) | | |
| Induction with a combination of A and T | 4 | (40) | | |
| Induction with T or T and Cytoxan | 2 | (20) | | |
| No. of prior regimens for metastatic disease | | | 1 | (1–2) |
| No. of induction chemotherapy cycles | | | 3 | (2–4) |
| Time from diagnosis to onset of metastasis, months | | | 30 | (0–117) |
| Time from onset of metastasis to HDCT, months | | | 4.9 | (3.8–24) |
| Prior radiation therapy to primary site | 1 | (10) | | |
| metastatic site | 1 | (10) | | |
| Radiation therapy post HDCT | 6 | (60) | | |
| Prior tamoxifen therapy as adjuvant | 4 | (40) | | |
| Hormonal therapy post-HDCT | 6 | (60) | | |

| **Sites of metastasis by patient** | **Response prior to HDCT** | **Response post HDCT** |
|---|---|---|
| Chest wall | CR (surgical) | cCR* |
| Lung | PR | CR |
| Lymph node | CR (surgical) | cCR |
| Bone | PR | cPR** |
| Lymph node | CR | cCR |
| Skin | CR | cCR |
| Bone | PR (radiated) | cPR |
| Lung/bone | PR/stable | cPR/SD |
| Skin/bone | CR/PR | cCR/cPR |
| Skin | CR (surgical) | cCR |

*Continuous CR; **continuous PR ±one patient received CMF followed by CAF.

In the larger group of patients who received 725 mg/m$^2$, no relationship could be identified between T pharmacokinetics and any of the laboratory parameters (ASAT, ALAT, bilirubin, alkaline phosphatase) tested; likewise, no correlation existed between T pharmacokinetics and either age or race (data not shown). We did observe however, that patients who weighed < 120% of their ideal body weight had significantly higher AUCs (82.2 μM×h (range, 59.9–96.1) vs. 54.8 μM×h (range, 26.2–91.5), $P$ = 0.04), higher $C_{max}$ (4.2 μM (range, 3.26–5.18) vs. 2.88 μM (range, 2.1–3.89), $P$ = 003), and lower clearances (10.3 l/h/m$^2$ (range, 8.83–14.2) vs. 15.5 l/h/m$^2$ (range, 9.27–32.4), $P$ = 0.04) than those patients weighing > 120% of their ideal body weight. T AUC ($P$ = 0.005), $C_{max}$ ($P$ = 0.01) and clearance ($P$ = 0.005) were better correlated with actual body weight than ideal body weight. However, in the case of AUC, when one very large patient was omitted from the analysis, the correlation became non-significant.

### Toxicity

### *Neurotoxicity*

Neurotoxicity was dose-limiting. The incidence of paraesthesias, nerve conduction abnormalities and grade 3 and 4 neurotoxicities is illustrated in Table 2. The incidence of paraesthesias vs. AUC is represented in Figure 3: while none of the patients treated at the first 2 dose levels, and less than 30% of patients treated at the first 8 dose levels of T reported paraesthesias of the hands and feet, reversible paraesthesias were reported by the majority of patients treated with T ≥ 575 mg/m$^2$. T dose levels were highly predictive for development of paresthesias ($P$ = 0.0002); greater T AUC (Figure 3) and peak concentrations (not shown) were also predictive for the development of paraesthesias but not more than T dose level (i.e. the amount of T/m$^2$ delivered; $P$ = 0.001 and $P$ = 0.0002 by Wilcoxon rank sum test, data not shown). Although nerve

 © 2001 Cancer Research Campaign

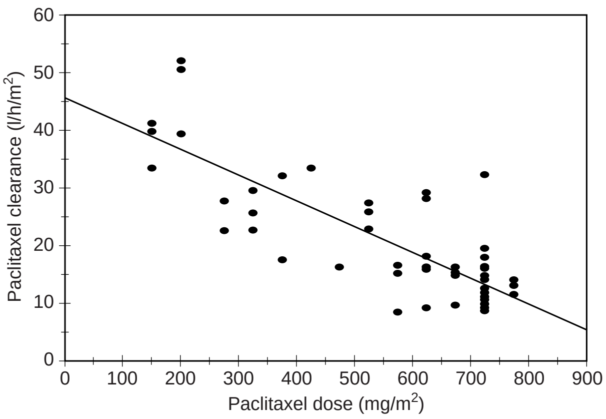

**Figure 2** Relationship between paclitaxel clearance and paclitaxel dose level, including line of regression

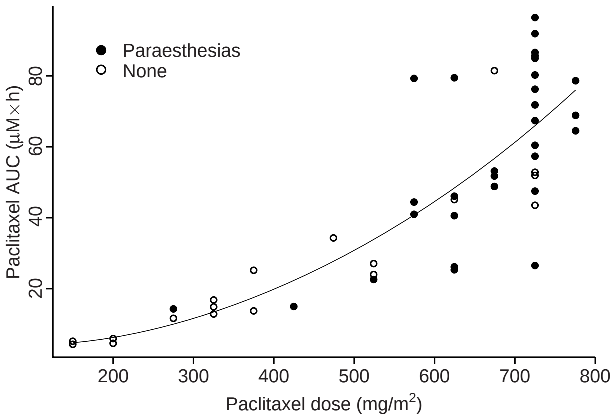

**Figure 3** Relationship between paclitaxel AUC, dose and incidence of paraesthesias. Line represents the quadratic equation that best describes the data. Closed symbols indicate patients with paraesthesias, while open symbols depict patients not reporting any neurotoxicity conduction studies were not available in all patients, more than half of those patients who had undergone such studies and were treated with T ≥ 625 mg/m$^2$ were found to have minimal to mild peripheral sensory and/or motor neuropathies. We have found no correlation between dose, peak concentration, or AUC and the presence of nerve conduction abnormalities. There was however, a trend correlating peak T concentration and dose-limiting grade 3/4 neurotoxicities ($P$ = 0.06); grade 3/4 peripheral neuropathies were associated with the highest dose levels of T ($P$ = 0.04, data not shown). The first grade 3 sensory neuropathy (difficulty with handwriting) was observed in a patient treated at T 625 mg/m$^2$. At T 675 mg/m$^2$ one patient developed grade 3 peripheral, primarily sensory, neuropathy manifesting as minimal gait and balance difficulties, with resolution of her symptoms in 5 months; a patient with pre-existing Charcot–Marie–Tooth disease also developed grade 3 sensory neuropathy manifesting as worsened handwriting; her symptoms resolved in 3 months. Due to her pre-existing neurological condition, we accured a seventh patient at the same dose level, who experienced no dose-limiting toxicities, hence T escalation continued. During T dose escalation 1 of 6 patients treated with T 725 mg/m$^2$ developed reversible, primarily sensory peripheral neuropathies. One patient treated with T 775 mg/m$^2$ developed a positive Romberg sign associated with grade 4 peripheral, primarily sensory neuropathy, hence defining the MTD; her symptoms slowly resolved in 8 months. The cohort of patients treated with T 725 mg/m$^2$ (MTD-1) was then expended to 20; 3 of these patients experienced grade 3 sensory/motor neuropathies.

### *Other toxicities*

There were no treatment-related deaths. Reversible grade 1/2 myalgias were observed in 1 patient each treated with T 150, 425, 475 and 525 mg/m$^2$ and in 4 patients treated at 725 mg/m$^2$. The severity and grade of mucositis, measured as the median number of days requiring narcotic analgesics (illustrated in Table 2) was independent of the dose, AUC or peak concentrations of T. The median number of days requiring hospitalization primarily due to mucositis and neutropenic fever was 15.5 (range, 8–25) (data not shown). There was no substantial decrease in LVEF as measured by MUGA scan following HDCT (see Table 2). However, one patient with prior radiation to the chest wall, and with subsequent excision of a subcutaneous metastasis above the sternum had a normal post-HDCT ejection fraction, but developed congestive heart failure and a LVEF of 25% after receiving additional radiation to the sternum. Her LVEF recovered to 45% with medical management and she is currently without symptoms. Haematopoietic toxicities were independent of T dose, AUC or peak concentrations. The median number of days to reach an absolute granulocyte count (AGC) of ≥ 0.5 × $10^9 l^{-1}$ and to reach platelet transfusion independence were 9 and 8, respectively (Table 2). One patient who had undergone mobilization with rhTPO and G-CSF developed acute lymphocytic leukaemia with 4/11 translocation (11q23) 12 months after HDCT. She achieved complete remission and is currently (18 months post induction) on maintenance therapy.

### Treatment response

With a median follow-up of 29 months (range 19–53 months) 30-month projected overall survival is 77% (95% CI, 64–93%) and relapse-free survival is 65% (95% CI, 51–83%) for patients with stage II and III breast cancer (see Figure 4). Of 10 patients treated for stage IV breast cancer 5 were in CR and 5 were in partial response, one of whom achieved a CR following HDCT. 4 of 10 patients treated for stage IV disease relapsed.

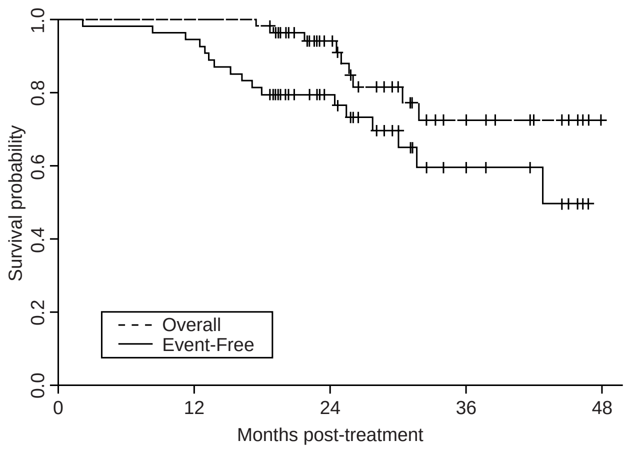

**Figure 4** Relapse-free and overall survival of patients with high-risk breast carcinoma following treatment with high-dose doxorubicin, cyclophosphamide and paclitaxel (ACT)

**Table 2** Taxol dose, neurological, gastrointestinal, haematopoietic toxicities and post-HDCT left ventricular ejection fraction EF

| Paclitaxel (mg/m²) | (N) | EF (%) post HDCT⁺ | Paraesthesias pts/cohort | Neurotoxicity 3 Grade 4 | Peripheral neuropathy* | | Days on narcotics** | Days to AGC > 0.5 × 10⁹L median (range) | Days to PLT & independence |
|---|---|---|---|---|---|---|---|---|---|
| 150 | (3) | 66 (57–69) | 0/3 | | N/A | | 5 (0–9) | 8 (7–9) | 8 (4–9) |
| 200 | (3) | 64 (62–67) | 0/3 | | N/A | | 11 (9–12) | 9 (8–10) | 8 (7–11) |
| 275 | (3) | 61 (52–66) | 1/3 | | minimal sensory/motor: | 1 | 11 (10–11) | 8 (8–11) | 7 (6–13) |
| | | | | | N/A: | 1 | | | |
| | | | | | none: | 1/3 | | | |
| 325 | (3) | 64 (62–66) | 0/3 | | mild sensory/motor: | 1 | 10 (8–15) | 10 (9–11) | 11 (5–14) |
| | | | | | none: | 2/3 | | | |
| 375 | (3) | 64 (57–72) | 1/3 | | minimal sensory/motor: | 1 | 9 (7–13) | 9 (9–10) | 9 (6–10) |
| | | | | | N/A: | 2 | | | |
| 425 | (3) | 66 (58–67) | 3/3 | | N/A: | 1 | 10 (7–11) | 8 (8–9) | 8 (8–10) |
| | | | | | none: | 2/3 | | | |
| 475 | (3) | 60 (56–83) | 1/3 | | N/A: | 3 | 5 (4–7) | 7 (7–9) | 6 (5–9) |
| 525 | (3) | 69 (56–83) | 1/3 | | Mild sensory/motor: | 1 | 11 (5–13) | 8 (8–8) | 7 (7–8) |
| | | | | | N/A: | 1 | | | |
| | | | | | Normal: | 1/3 | | | |
| 575 | (3) | 77 (65–77) | 3/3 | | mild motor: | 11 | 12 (12–19) | 9 (8–9) | 8 (8–9) |
| | | | | | N/A: | 2 | | | |
| 625 | (6) | 58 (51–63) | 5/6 | 1 | mild sensory: | 1 | 6.5 (0–11) | 9 (7–10) | 6.5 (5–9) |
| | | | | | mild motor: | 2 | | | |
| | | | | | delayed/missing H reflex:≠ | 3 | | | |
| | | | | | N/A: | 1 | | | |
| | | | | | none: | 1/6 | | | |
| 675 | (7) | 61 (53–68) | 5/7 | 2+ | minimal sensory/motor: | 1 | 9 (0–20) | 10 (8–10) | 7 (6–10) |
| | | | | | mild sensory/motor: | 2+ | | | |
| | | | | | prolonged H reflex: | 1 | | | |
| | | | | | N/A: | 1 | | | |
| | | | | | none: | 2/7 | | | |
| 725 | (20) | 63 (50–86) | 16/20 | 3 | mild sensory/motor: | 6 | 10 (0–14) | 9 (6–12) | 7 (3–11) |
| | | | | | mild motor: | 3 | | | |
| | | | | | delayed/missing H reflex: | 2 | | | |
| | | | | | N/A | 6 | | | |
| | | | | | none: | 5/20 | | | |
| 775 | (3) | 67 (62–71) | 3/3 | 1 | mild sensory/motor: | 2 | | | |
| | | | | | mild motor: | 1 | 12 (7–15) | 9 (9–9) | 9 (9–9) |

⁺: MUGA scans were performed within 30 days from HDCT; *: Combined, predominantly sensory and motor peripheral neuropathy as documented by nerve conduction studies of peroneal, posterior tibial and sacral nerves; **: Prescribed to alleviate pain due to mucositis; + One patient was treated with pre-existing Charcot-Marie–Tooth disease; & Platelet independence is defined as the day after the last platelet transfusion; ≠: monosynaptic reflex with an afferent/efferent limb.

*© 2001 Cancer Research Campaign*

### DISCUSSION

The benefit of standard dose T as a 3-hour i.v. infusion subsequent to adjuvant doxorubicin and cyclophosphamide in patients with HRBC is unclear. The shorter infusion time may be responsible for lack of clear benefit in HRBC (Henderson et al, 1998), and results with such schedule are inferior in the metastatic setting (Mamounas et al, 1998). High-dose 24-hour infusion of T was first tested as a component of HDCT in stage IV disease. Escalating doses of T with cyclophosphamide and cisplatin resulted in a 54% overall response rate; severe sensory neuropathy and central nervous system toxicity beyond T 775 mg/m$^2$ were limiting (Stemmer et al, 1996). Severe peripheral neuropathy was observed with > T 775 mg/m$^2$ (Vahdat et al, 1998) when T was administered over 24-hours as part of a triple HDCT regimen, followed first by melphalan and subsequent cyclophosphamide, thiotepa and carboplatin (STAMP V); this combination yielded a 74% overall response rate. However, neurotoxicity was not reported when T 250 mg/m$^2$ was infused over 3 hours as part of HDCT with carboplatin and cyclophosphamide, but, 2 of 21 HRBC patients died on day 10, following reinfusion of CD34$^+$ selected PBPC (Greinix et al, 2000).

Since the majority of patients on our trial of ACT were treated for HRBC, for the sake of safety we chose to escalate the dose of T very carefully. We observed a diminished degree of mucositis and haematopoietic toxicity compared to our experience with CAVP. No patient treated with T 725 mg/m$^2$ (the proposed phase II dose) experienced grade 3 mucositis, versus 26% of patients treated with our prior CAVP regimen (data not shown). Patients treated at the MTD-1 dose of T 725 mg/m$^2$ required hospitalization for mucosities and neutropenic fever for a median of 15.5 days (range, 8–25) versus 29 days for patients treated with CAVP (data not shown). Granulocyte recovery was similar following ACT versus CAVP at this dose level, but platelet independence was achieved earlier with ACT (data not shown). As for cardiac tolerance: a recent phase I trial of a cyclophosphamide, mitoxantrone and 3–6 hour infusion of T was associated with severe cardiac toxicities above T 400 mg/m$^2$; a 24-hour schedule seems preferable (Gluck et al, 1998).

T pharmacokinetics were predictive of haematopoietic toxicities, mucositis and neurotoxicities at conventional dose levels (Brown et al, 1991; Wilson et al, 1994; Gianni et al, 1997); we observed such correlations only between dose, peak concentration, AUC and development of paraesthesias. The dose-limiting toxicity at T 775 mg/m$^2$ was reversible peripheral neuropathy. Peak concentrations and AUC of T were no more predictable for neurotoxicities then knowing the prescribed dose of T; the relatively small sample size and wide interpatient pharmacokinetic variability might explain such phenomena.

The observed relapse-free survival in patients with HRBC is in keeping with our previous cohort of CAVP-treated patients at this length of follow-up. The cohort of patients treated for stage IV disease is too small to assess the value of this regimen in that setting.

In conclusion, high-dose chemotherapy with doxorubicin, cyclophosphamide and infusional T (ACT) is feasible, but mucositis necessitating the use of narcotic analgesics, reversible peripheral neuropathy, and the requirement for hospitalization represent significant toxicities. T pharmacokinetics become non-linear at higher dose levels with increasing interpatient variability, suggesting saturable tissue distribution and/or elimination. Higher doses of T are associated with an increased incidence of reversible peripheral neuropathy. The potential therapeutic benefit and manageable toxicity profile of ACT needs to be further evaluated. Preliminary data from a completed, randomized US, trial did not reveal a survival benefit in patients with primary breast cancer, possibly due to unacceptably high regimen-related mortality (Peters et al, 1999); no data are available from another completed, randomized US trial, while encouraging data from the Rodenhuis trial should be considered preliminary (Rodenhuis et al, 2000). In the metastatic setting equivalence of the STAMP V regimen to prolonged administration of CMF was noted in patients in partial remission (Stadtmauer et al, 2000), while a smaller trial suggested improved relapse-free but equivalent overall survival following HDCT versus conventional treatment (Lotz et al, 1999). We are currently in the process of comparing the toxicity and efficacy of ACT against the STAMP V regimen in women with high-risk primary breast cancer. Should the final results of completed randomized studies confirm the beneficial role of HDCT in the treatment of selected patients, the less toxic, and at least equally effective regimen can later be tested in a larger phase III randomized trial.

### ACKNOWLEDGEMENTS

We thank Judy Brent, Melissa Scalia and Tricia Taylor for their assistance with the clinical aspects and data management and preparation of this manuscript. This study was supported by grants CA33572 and CA62505 from the National Cancer Institute Cancer Center and by Bristol-Myers Squibb Corporation.

### REFERENCES

Antman KH, Rowlins PA, Vaughan WP, Pelz CJ, Fay J, Fields K, Freytes C, Gale RP, Hillner B, Holland K, Kennedy J, Klein J, Lazarus H, McCarthy P, Saez R, Spitzer G, Stadtmauer E, Williams S, Wolff S, Sobocinski K, Armitage J and Horowitz M (1997) High-dose chemotherapy with autologous hematopoietic support for breast cancer in North America. *J Clin Oncol* **15**: 1870–1879

Bronchud MH, Howell A, Crowther D, Hopwood P, Souza L and Dexter TM (1989) The use of granulocyte colony-stimulating factor to increase the intensity of treatment with doxorubicin in patients with advanced breast and ovarian cancer. *Brit J Cancer* **60**: 121–125

Brown T, Havlin K, Weiss G, Cagnola J, Koeller J, Kuhn J, Rizzo J, Craig J, Phillips J, and Von Hoff D (1991) A phase I trial of Taxol given by 6-hour intravenous infusion. *J Clin Oncol* **9**: 1261–1267

Doroshow JH, Somlo G, Ahn C, Baker P, Rincon A, Forman S, Akman S, Chow W, Coluzzi P, Hamasaki V, Leong L, Margolin K, Molina A, Morgan R, Rashko J, Shibata S, tetef M, Yen Y and Brent J (1995) Prognostic factors predicting progression-free (PFS) and overall survival (OS) in patients (PTS) with responsive metastatic breast cancer (MBC) treated with high-dose chemotherapy (HDCT) and bone marrow stem cell reinfusion. *Proceedings of Asco* **Vol. 14.** pp.319: Los Angeles, CA

Gianni L, Kearns CM, Gianni A, Capri G, Vigano L, Lacatelli A, Bonadonna G and Egorin MJ (1995) Nonlinear pharmacokinetics and metabolism of paclitaxel and its pharmacokinetic/pharmacodynamic relationships in humans. *J Clin Oncol* **13**: 180–190

Gianni L, Vigano L, Locatelli A, Capri G, Giani A, Tarenzi E and Bonadonna G (1997) Human pharmacokinetic characterization and in vitro study of the interaction between doxorubicin and paclitaxel in women with breast cancer. *J Clin Oncol* **15**: 1906–1915

Gluck S, Germond C, Lopez P, Cano P, Doreen M, Koski T, Arnold A, Dulude H and Gallant G (1998) A phase I trial of high-dose paclitaxel, cyclophosphamide and mitoxantrone with autologous blood stem cell support for the treatment of metastatic breast cancer. *Eur J Cancer* **34**: 1008–1014

Greinix H, Linkesh W, Seifert M, Kubista E, Czerwenka K, Elahi F, Zielinski C, Hoecker P, Steger G, Schulenberg A, Neumeister G, Rabitsch W, Jakesz R and Kalhs P (2000) Paclitaxel-containing high-dose chemotherapy in high-risk breast cancer patients *Acta Oncol* **39**: 47–52

Henderson I, Berry D, Demetri G, Cirrincione C, Goldstein L, Martino S, Ingle J, Cooper M, Canellos G, Borden E, Fleming G, Holland J, Graziano S, Carpenter J, Muss H < Norton L for CALGB, ECOG, SWOG and NCCTG (1998) Improved disease-free and overall survival from the addition of sequential paclitaxel but not from the escalation of doxorubicin dose level in the adjuvant chemotherapy of patients with node-positive primary breast cancer. *Proceedings of Asco* **Vol. 17.** Pp.101a: Los Angeles, CA

Holmes FA, Walters RS, Theriault RL, Forman AD, Newton LK, Raber M, Buzdar A, Frye D and Hortobagyi GN (1991) Phase II trial of taxol, an active drug in the treatment of metastatic breast cancer. *J Natl Cancer Inst* **83**: 1797–1805

Hortobagyi G, Buzdar A, Theriault R, Valero V, Booser D, Frye D, Holmes F, Giralt S, Khouri I, Andersson B, Gajewski J, Rondon G, Smith T, Singletary SE, Ames F, Sneige N, Strom E, McNeese M, Deisseroth A and Champlin R (2000) Lack of efficacy of adjuvant high-dose tandem combination chemotherapy for high-risk primary breast cancer – A randomized trial. *J Natl Cancer Inst* **92**: 225–233

Kaplan EL, Meier P: Nonparametric estimation from incomplete observations (1958) *J Am Stat Assoc* **53**: 457–481

Lotz JP, Cure H, Janvier M, Morvan F, Legros M, Asselain B, Guillemot M, Roche H and Gisselbrecht C (1999) [Intensive chemotherapy and autograft of hematopoietic stem cells in the treatment of metastatic cancer: results of the national protocol Pegase 04]. Intensification therapeutique et autogreffe de cellules souches hematopoietiques (CSH) dans le traitment des cancers du sein metastatiques: results du programme national Pegase 04. *Hematol Cell Ther* **41**: 71–74

Mamounas E, Brown A, Smith R, Lembersky B, Fisher B, Wickerham D, Wolmark N, Atkins J, Shibata H, Baez L, DeFusco P, Davila E, Thirwell M, Bearden J, Tipping A and Scholnick A (1998) Effect of taxol duration of infusion in advanced breast cancer: Results from NSABP B-26 trial comparing 3- to 24 hour infusion of high-dose taxol. *Proceedings of Asco* **Vol.17.** pp. 101a: Los Angeles, CA

McCloskey DE, Kaufman SH and Prestigiacomo LJ: (1996) Paclitaxel induces programmed cell death in MDA-MB-468 human breast cancer cells. *Clin Cancer Res* **2**: 847–854

O'Shaughnessy JA, Fisherman JS and Cowan KH (1994) Combination paclitaxel (Taxol) and doxorubicin therapy for metastatic breast cancer. *Sem Oncol* **21**: 12–23

Peters WP, Ross M, Vredenburg JJ, Meisenberg B, Marks LB, Winer E, Kurtzberg J, Bast RC Jr, Jones R, Shpall E, Wu K, Rosner G, Gilbert C, Mathias B, Coniglio D, Petros W, Henderson IC, Norton L, Weiss RB<Budman DR and Hurd D (1993) High-dose chemotherapy and autologous bone marrow support as consolidation after standard-dose adjuvant therapy for high-risk primary breast cancer. *J Clin Oncol* **11**: 1132–1143

Peters W, Rosner G, Vredenburgh J, Shpall E, Crump M, Richardson P, Marks L, Cirrincione C, Wood W, Henderson I, Hurd D, Norton L for CALGB, SWOG and NCIC (1999) A prospective, randomized comparison of two doses of combination alkylating agents (AA) as consolidation after CAF in high-risk primary breast cancer involving ten or more axillary lymph nodes (LN): preliminary results of CALGB 9082/SWOG 9114/NCIC MA-13. *Proceedings of Asco* **Vol.18.** Pp.1: Atlanta, GA

Reichman BS, Seidman AD, Crown JPA, Heelan R, Hakes R, Lebwohl D, Gilewski T, Surbone A, Currie V and Hudis CA, Yao T, Klecker R, Jamis-Dow C, Collins J, Quinlivan S, Berkery R, Taomasi F, Canetta R, Fisherman J, Arbuck S and Norton L (1993) Paclitaxel and recombinant human granulocyte-colony stimulating factor as initial chemotherapy for metastatic breast cancer. *J Clin Oncol* **11**: 1943–1951

Riley CA, Crom WR and Evans WE (1985) Loop-column extraction and liquid chromatographic analysis of doxorubicin and three metabolites in plasma. *Therapeutic Drug Monitoring* **7**: 455–460

Rizzo J, Riley C, Von Hoff D, Kuhn J, Phillips J and Brown T (1990) Analysis of anticancer drugs in biological fluids: Determination of Taxol with application to clinical pharmacokinetics. *J Pharmaceut Biomed Anal* **8**: 159–164

Rodenhuis S, Richel D, van der Wall E, Schornagel J, Baars J, Koning C, Peterse J, Borger J, Nooijen W, Bakx R and Dalesio O (1998) A randomized trial of high-dose chemotherapy and hematopoietic progenitor cell support in operable breast cancer with extensive axillary lymph node involvement. *Lancet* **352**: 515–521

Rodenhuis S, Bontenbal M, Beex L, van der Wall E, Richel D, Nooij M, Voest E, Hupperets P, Westermann A, Dalesio O and de Vries E (2000) Randomized phase III study of high-dose chemotherapy with cyclophosphamide, thiotepa and carboplatin in operable breast cancer with 4 or more axillary lymph nodes (2000) *Proceedings of Asco* **Vol. 19**. Pp. 74a: New Orleans, LA

Somlo G, Doroshow JH, Forman SJ, Leong L, Margolin K, Morgan R Jr, Raschko J, Akman S, Ahn C, Nagasawa S, Harrison J, Stein A, Smith E, Sniecinski I and Schmidt G (1994) High-dose doxorubicin, etoposide and cyclophosphamide with stem cell reinfusion in patients with metastatic or high-risk primary breast cancer. *Cancer* **73**: 1678–1685

Somlo G, Doroshow JH, Forman SJ, Odom-Maryon T, Lee J, Chow W, Hamasaki V, Leong L, Morgan R Jr, Margolin K, Raschko J, Shibata S, Tetef M, Yen Y, Simpson J and Molina A (1997a) High-dose chemotherapy and stem cell rescue in the treatment or high-risk breast cancer: Prognostic indicators of progression-free and overall survival. *J Clin Oncol* **15**: 2882–2893

Somlo G, Sniecinski I, Odom-Maryon T, Nowicki B, Chow W, Hamasaki V, Leong L, Margolin K, Morgan Jr. R, Raschko J, Shibata S, Tetef M, Molina A, Berenson R, Forman S and Doroshow JH (1997b) The effect of CD34 + selection and various schedules of stem cell reinfusion and priming on hematopoietic recovery following high-dose chemotherapy for breast cancer. *Blood* **89**: 1521–1528

Sonnichsen D, Hurwitz C, Pratt C, Shuster J and Relling MV (1994) Saturable pharmacokinetics and paclitaxel pharmacodynamics in children with solid tumors. *J Clin Oncol* **12**: 532–538

Stadtmauer E, O'Neill A, Goldstein L, Criley P, Mangan K, Ingle J, Brodsky I, Martino S, Lazarus H, Erban J, Sickles C, Glick J and the Philadelphia Transplant Group (2000) Conventional-dose chemotherapy compared to high-dose chemotherapy plus autologous hematopoietic stem-cell transplantation for metastatic breast cancer *N Engl J Med* **342**: 1069–1076

Stemmer S, Cagnoni PJ, Shpall EJ, Bearman S, Matthes S, Dufton C, Day T, Taffs S, Hami L, Martinez C, Purdy M, Arron J and Jones RB (1996) High-dose paclitaxel, cyclophosphamide and cisplatin with autologous hematopoietic support: a phase I trial. *J Clin Oncol* **14**: 1463–1472

Synold TW and Doroshow JH (1996) Anthracycline dose intensity: clinical pharmacology and pharmacokinetics of high-dose doxorubicin administered as a 96-hour continuous intravenous infusion. *J Infusional Chemother* **6**: 69–73

Vahdat LT, Papadopuoloski K, Balmaceda C, McGovern T, Dunleavy J, Kaufman E, Fung B, Garrett T, Savage D, Tiersten A, Ayello J, Bagiella E, Heitjan D, Antman K and Hesdorffer C (1998) Phase I trial of sequential high-dose chemotherapy with escalating dose paclitaxel, melphalan and cyclophosphamide, thiotepa and carboplatin with peripheral blood progenitor support in women with responding metastatic breast cancer. *Clin Cancer Res* **4**: 1689–1695

Williams SF, Mick R, Desser R, Golick J, Beschorner J and Bitran J (1989) High-dose consolidation therapy with autologous stem cell rescue in stage IV breast cancer. *J Clin Oncol* **7**: 1824–1830

Wilson WH, Berg SL, Bryant G, Wittes R, Bates S, Fojo A, Steinberg S, Goldspiel B, Herdt J, O'Shaughnessy J, Balis F and Chabner B (1994): Paclitaxel in doxorubicin-refractory or mitoxantrone-refractory breast cancer: A phase I/II trial of 96 hour infusion. *J Clin Oncol* **12**: 1621–1629

Wood WC, Budman DR, Korzun AH, Cooper M, Younger J, Hart R, Moore A, Ellerton J, Norton L, Ferree C, Ballow A, Frei E III and Henderson I (1994) Dose and dose intensity of adjuvant chemotherapy for stage II, node-positive breast carcinoma. *N Engl J Med* **330**: 1253–1259

*© 2001 Cancer Research Campaign*
